# Co-designing a film showcasing the dental experiences of community returners (ex-offenders)

**DOI:** 10.3389/froh.2024.1391438

**Published:** 2025-01-06

**Authors:** Joelle Booth, Heather McMullen, Andrea Rodriguez, Vanessa Muirhead

**Affiliations:** ^1^ Centre for Dental Public Health and Primary Care, Institute of Dentistry, Queen Mary University of London, London, England; ^2^ Peninsula Dental School, Faculty of Health, University of Plymouth, Plymouth, England; ^3^ Centre for Public Health and Policy, Wolfson Institute of Population Health, Queen Mary University of London, London, England; ^4^ School of Dentistry, University of Dundee, Dundee, Scotland

**Keywords:** community research, patient and public partnership, co-creation, co-design, catalyst film, participatory action research, arts-based research methods, social exclusion

## Abstract

**Background:**

The oral health of over 90,000 individuals in UK prisons is four times worse than the general population. A recent scoping review on the oral health of prisoners inside the justice system highlighted the lack of research about what happens when they transition out of prison to become community returners.

**Objectives:**

To co-design a film to showcase the dental experiences of community returners before and after they transition out of prison, change perceptions and inform oral health research priorities.

**Methods:**

This action research involved five community returners, recruited through third sector organisations, who attended virtual workshops. Participants in the first workshop designed the storyboard; community returners incorporated their own stories into fictional characters to portray their lived experiences. They developed the character stories and wrote the script in the second workshop. A community film production company produced the film and used professional actors who had contact with the justice system to depict the characters in the film.

**Results:**

The final film, titled “My Story, My Words, My Mouth” explored themes such as self-care oral health behaviours, dental care provision in prison, access to healthcare, stigmatisation, disclosure and improving oral health to support societal reintegration. The film was screened at an open event for stakeholders and included a question-and-answer session and recorded videos where viewers shared their feedback to inform future research projects.

**Conclusion:**

Co-design can be an empowering platform to hear the voices of community returners. Using the medium of film an oral health promotion tool can build understanding about the oral health needs of underrepresented groups. This egalitarian and power-sharing approach can also provoke critical discussion and actively involve underrepresented people in research that impacts their lives to develop strategies, to set priorities and improve their oral health.

## 1 Introduction

Release from prison is a challenging time for ex-offenders, more positively referred to as community returners (1). Community returners face substantial health inequalities with higher mortality and morbidity risks than the general population (2–5). People who have experienced incarceration also have higher levels of mental health disorders, higher rates of suicide (6, 7) and those within prison experience dental decay at four times the rate of the general population (8). The transition period for people struggling with substance misuse issues can also make them increasingly vulnerable to overdose or relapse (9, 10). Despite these inequalities, community returners are less likely to access health services because of multiple barriers (11). Some of the reasons cited in the Care for Offenders Continuity of Access report are; past experiences of breakdowns in trust, being homeless, having a disordered lifestyle, long waiting times to be seen by healthcare services, negative perceptions of healthcare and a lack of flexibility from healthcare services (12, 13). Freeman et al. found in their qualitative research that community returners often do not access dental services because of fear of being judged or discriminated against by dental healthcare professionals (14). However, contact with the criminal justice system provides an opportunity for individuals to engage with healthcare and adopt health promoting behaviours while they are in prison in many countries (15). As oral health plays an integral role in an individual's self-confidence, ability to socialise, seek employment and their overall wellbeing, good oral health is essential in supporting community returners to successfully navigate life on release from prison (16–18). While there is some evidence about access to dental services for people in prison, there is a paucity of knowledge about the perceptions community returners have of accessing dental care on release and the feelings about their own oral health.

Film is an impactful medium that can be used as an alternative methodology to convey the complex intricacies of an individual's lived experience. Film has been used in several ways within health research to portray complex concepts, advocate for social change, capture current perceptions, promote discussion around sensitive topics and engage socially excluded populations (19, 20). Whilst film has predominantly been used within disciplines such as sociology and anthropology (21) this powerful methodology is translating through to health and dental research with the improvement of digital accessibility further enhancing this shift (19, 22, 23). Interestingly, a scoping review exploring the use of film in public health research showed that a quarter of the studies using film within their methodology did so to explore sensitive topics suggesting that this is an appropriate methodology to facilitate discussion around socially sensitive issues (23). Storytelling is an effective tool that can help raise awareness of public health messaging, advocate for community change and present a collective voice on complex issues which why it was selected as the methodology of choice to convey the dental experiences of community returners (24, 25).

Co-design is a process by which active collaborations are formed with stakeholders to improve or solve predefined health problems (26). A co-design approach acknowledges the value individuals with lived experience can bring to research (27). Those with a lived experience are best placed to guide research and shape health services to improve their health. Co-design uses a collaborative approach that requires a philosophical shift away from a traditional research hierarchy towards a shared decision-making model (28). The principle of co-design rests on actively involving those with lived experience to ensure that the end outcome meets their needs, expectations and has meaningful impact. It is believed that using a co-design approach can reduce research waste through narrowing the gap between the perspective of researchers and the expectations of the communities they serve (29). Furthermore, co-design can act as a powerful inclusion health tool to engage populations that have previously been “othered” and faced exclusion, with research being conducted about them rather than with or alongside them (30). The degree to which co-design is used can vary greatly from participation at the research design stage alone through to continued engagement throughout the entirety of a research project. With this, the aim of co-design varies, and it can be used as a tool to set research priorities, contribute to protocols, direct study design, or contribute to outcomes such as health education material (31). However, it is vital that co-design is embedded within research methodologies rather than being considered at a later stage or used as tokenistic engagement.

This paper describes a unique participatory action research approach used to co-design a film addressing the highly relevant oral health experiences of community returners. The aim of this paper is to showcase how a co-design approach can be used to create a catalytic film to bring the voices of those with a lived experience of the criminal justice system to the forefront. We describe the process of utilising connections with third sector organisations for recruitment, establishing power-sharing dynamics, providing participants with opportunities to develop new skills in script writing and using the medium of film to share lived experiences. This process is an example of how co-design can be used to facilitate inclusive collaborations to integrate the voices of those with a lived experience of the criminal justice into oral health research.

## 2 Methods

This patient and public involvement and engagement (PPIE) project utilised a co-design approach with the aim of creating a catalyst film to showcase the oral health experiences of community returners. A participatory action research methodology was used, community returners were empowered to stimulate change through the medium of film to improve awareness of the oral health challenges they have faced due to their history of contact with the criminal justice system (32). Participatory action research focuses on the purpose of enabling action, for this study the action output was the development of a film to showcase their lived experiences with the aim of stimulating conversation, raising awareness, and informing future research priorities. This approach was selected as it allowed participants to contribute data, in this case their lived experience which formed the concepts for the film, analyse these and then decide on which action should follow by way of determining the film content. This process was repeated as the initial outline of the film allowed for reflection and adaptation of the content by the participants, see Supplementary Information (S1). Participatory action research requires the researchers to have a conscious awareness of pre-existing power relationships and actively advocate for power to be shared with the community returners. This notion was fundamental to the ethos of this project and underpinned the design and execution of the study.

The Queen Mary University of London Research Ethics Committee (REC) were consulted, and ethical approval was not required as this was an engagement project (32). However, ethical standards were adhered to throughout with participants being sent participant information sheets prior to joining the project and signing informed consent forms (33). The upmost care was taken to ensure the confidentiality of those participating to allow them to have a safe space to freely express their views and share their experiences. Participants were offered the option to meet with the research team prior to joining the project and given the opportunity for the participant information sheet to be verbally explained to them prior to deciding whether they would like to take part in the project. In addition to this, the research team were mindful throughout the project of the importance of ensuring that the experiences generously shared by community returners were portrayed sensitively, respectfully and were an accurate representation of their lived experiences.

### 2.1 Recruitment

Community returners were recruited to join the project and co-design the film storyboard and script. Recruitment began in January 2023 and the research team used multiple approaches to present the opportunity to a range of community returners. There is a lack of information as to the best methodologies to use to recruit those who have had contact with the criminal justice system so to combat this several methods were used. Initially, third sector community partners (community and voluntary sector organisations) were used as gatekeepers to this cohort. There are several third sector groups who work closely with community returners to offer support in numerous ways such as providing employment opportunities, influencing criminal justice policy, offering mentoring, and supporting the families of people within the criminal justice system. Third sector organisations were contacted, provided with a brief of the project, and asked if they would be able to aid recruitment. The organisations varied in how they supported recruitment, some were able to reshare a recruitment advert on their established social media pages, others published the opportunity in their newsletters disseminated to those they support and a couple of organisations hand selected individuals through support workers that they felt would be interested in the opportunity. In addition to recruiting through third sector groups, a flyer advert was created which was shared on social media. A snowballing approach was also used by which those who were interested in taking part in the project were welcomed to invite peers.

### 2.2 Workshop one—identifying oral health issues

The aim of the first workshop was to bring community returners together to discuss which topics surrounding oral health they wanted to depict in the film and how they wanted to present them. The research team met prior to the workshop to outline the first workshop structure. Both workshops were hosted virtually via Microsoft Teams and participants were given a £75 voucher for each half day workshop they attended. This monetary value was set to adhere with the National Institute for Health and Care Research's payment guidance for researchers and professionals (34). The voucher type chosen was selected as it allowed participants the most freedom in where they were able to use the voucher. It was decided that the workshops would be hosted virtually to give attendees the option of using pseudonyms and keeping their cameras turned off so that they had the option of remaining anonymous to other attendees. Special consideration was taken to ensure that potential participants were not digitally excluded by providing the option of paying for data to allow attendees to take part using a mobile phone if they did not have access to a laptop or the internet. Consent was taken from the participants to record both workshops through Microsoft Teams. The purpose of recording the workshops was to ensure that the community returner contributions were accurately recorded in the film contents and script. Once the script had been finalised these recordings were deleted and none of the content was formally thematically analysed. Outputs were also documented by a member of the research team present at each workshop who acted as a notetaker.

Prior to workshop one an online pack was sent to attendees Supplementary Information (S2). The pack included a brief overview of the project, the agenda for the workshop, examples of previous films created by the film production organisation and another film discussing prison health (35). These resources were sent along with thought-provoking questions about the potential styles and storyboards that could be used for the film. The storyboards were used as a prompt for discussion in the workshops as well as being a necessary step required in creating a film. The pre-workshop pack helped to communicate with participants what to expect in the first workshop and set clear aims for the workshop.

During the first workshop a short presentation was given by the research team to attendees to introduce the project, the research team, and the aim of workshop one. It was emphasised that the aim of the first workshop was to determine which issues the community returners felt were important to present to the audience, the style of film they wanted and to outline the storyboard for the film. Ground rules were established early in the workshop following the introductions. The emphasis for these ground rules were on ensuring participants understood that this was a co-design project, all opinions are equally valued, the thoughts of others respected even if they differ from that of another participant and that personal experiences shared in the workshops should remain confidential. The only exception to the rule of confidentiality was in the case of any adaptations of experiences individuals volunteered to be put forward to be included in the film storyboard or script. Establishing these ground rules helped to facilitate an environment in which everyone involved with the project felt able to communicate their thoughts knowing that their ideas would be respected and valued. The workshop lasted half a day and was facilitated by two members of the research team.

### 2.3 Workshop two—telling the stories

The second workshop was held one month following the first workshop and all those who attended the first workshop were invited to join. As with the first workshop a pre-workshop pack was sent to attendees via email, this pack was put together by the research team and included aspects for the group to consider suggested by the film production organisation (S2). The pre-workshop pack for workshop two contained the aim for the workshop, the agenda, a summary of the storyboard that was decided in workshop one and the outline of the characters that would feature in the film based on the discussions from workshop one. The aim of the second workshop was to write the script for the two community returner characters presented in the film. To best facilitate the co-writing exercise the group was split into two with three participants writing the content for one character and the remaining two participants writing the script for the second character. Each of these groups were facilitated by a researcher who acted as a scribe to document the scripts written by the participants.

### 2.4 Filming

The filming, production and editing of the film was conducted by the community organisation, Mile End Community Project (MCP) (36). MCP teaches film production to young people living in a deprived area of East London to equip them with the skills needed to better their own lives and the lives of those in their community. This organisation was identified through their existing connections with the Queen Mary University of London Public Engagement team who funded this project (37). Following the first workshop a member of the research team met with the film production company to discuss the storyboard for the film. Feedback was given by the film production company, Mile End Community Project, on the proposed storyboard design and the logistical aspects of capturing the desired content. This feedback was relayed to the community returners and considered when writing the script during the second workshop. The film production company were able to utilise their experience of working on previous community film projects to offer advice on aspects relating to filming. Two professional actors were hired through Synergy Theatre Project, an organisation that provides practical art experiences for community returners, individuals in prison, and those at risk of offending (38). As the two actors both had a lived experience of the criminal justice system, they were sent the scripts in advance and offered the opportunity to make adaptations to reflect their own experiences of being community returners. All filming was completed over the course of a single day in a variety of locations including a film studio, outside a tube station and in a dental hospital. Once filming was completed and the first draft was edited the research team offered their feedback to ensure the edit accurately reflected the content of the workshops whilst meeting the aims of the project. Evidence based facts published in existing academic literature were added to the film to provide a context to the character stories and included prior to the credits.

The final film was then viewed by the research team who identified six main themes and issues raised through the film by the community returners. These themes related both to historical factors that impacted the oral health of community returners and elements of their life on release that made achieving good oral health difficult. To illustrate each of these themes for consideration, quotes were extracted from the film transcript created by the film production company to add closed captions to the film. These themes and quotes are presented in Table 1 of the results section.

**Table 1:** Themes explored in the film and associated reference in the film.

Themes explored in the film	
Themes explored	Reference in the film
Self-care oral health behaviours	“I would say the motivation, to look after myself changed, and everything's so expensive. You know, like I make, I make two pound a day. So when it comes to making a decision, do I buy toothpaste or do I buy phone credit? Speak to my family. It's…it's a hard decision to make.”
Impact of poor oral health on general health	“Having all those teeth removed, screwed up my digestion.”
Dental care provision in prison	“When I first came in, I put a request in to see the dentist. I had this excruciating pain in one of my teeth and just nothing happened. So, I just had to spend the time alone in the cell in agony for 18 months and then they moved us, I moved prison. So, I had to start the whole thing all over again. Right back to the bottom of a brand new list.”
Stigmatisation	“I called up my old dentist. It took me weeks cuz I was, I was terrified. I was terrified of, what do you think? You know, where had I been? Or did he know where I was? If he did, what did he think? Cause I think about it, a lot. I think about going back a lot.”
The role oral health plays in societal reintegration	“After lots of struggle and lots of dental appointments, I finally managed to get my teeth fixed. I'm at a better place now where I'm happy with my teeth and I'm confident to smile.”
Community returners’ experiences of not being able to access their prison health records after they returned to community settings, leading to embarrassment at having to disclose their prison histories to healthcare staff	Receptionist: “I'm really struggling to find you on the system. What was your last address?” Male character: “Um HMP… HMP Durham”.

## 3 Results

The results of this project are presented in line with the key principles outlined in the National Institute for Health and Care Research (NIHR) Learning for Involvement Guidance for Co-producing a Research Project (39).

The content for the film was derived from suggestions provided by the community returner group and the lived experiences they shared with the group; this content has been summarised in Table 1. Five community returners were recruited, three females and two males. The community returners varied in how long they had been in prison, the categories of prisons they had stayed in, number of sentences served and time since release. During the first workshop during which the structure of the film was determined it became apparent that the community returners had a range of different oral health experiences. For example, some of the participants had been very motivated with strict oral health and wider health promoting behaviours prior to being convicted. Other members of the group reflected on how they had competing priorities prior to conviction and looking after their teeth was not *a priori*ty for them. This meant that whilst some individuals had entered prison with good oral health and having previously regularly accessed dental care, this was not the case for most of the group.

Although the focus of the film was to depict oral health experiences on release from prison, the group wanted to convey their oral health experiences whilst in the prison system and felt this was important to help the viewer to understand the challenges they experience on release. All participants spoke about the barriers they experienced in accessing regular dental care whilst in prison and one of these experiences is presented in the final film. Barriers discussed included being unable to get to dental appointments due to prison lockdown protocols, long waiting lists to get a dental appointment or dentists only seeing emergency cases and not offering routine care. The group also referred to the effects reduced access to dental care in prison had on their oral health on release. These implications extended to the wider consequences of poor oral health on their overall wellbeing such as impacting nutritional intake and contributing to decreased self-confidence.

Participants wanted to capture in the film how a community returner might feel accessing dental care for the first time on release. Individuals discussed the challenges around the practicality of obtaining a dental appointment when they are often no longer ‘registered’ to a dental practice or might have relocated to a new geographical area since their release. For those who returned to the same location on release they felt apprehensive about returning to a dental practice where the staff could be aware of their contact with the justice system. A common theme raised was how overwhelming exposure to busy areas can be and how challenging it can be to navigate getting to dental appointments. Both concepts were explained by the group as being due to the contrast from their confined and regulated lives in prison. They spoke about become accustomed to the sounds and daily routine of prison life and how different this is to their lives is on release as they re-gain their autonomy.

On the theme of disclosure, some of the participants shared with the group how they felt they needed to explain to dental professionals why their teeth had become so bad by disclosing their history of incarceration. Others did not want to disclose their criminal justice histories but felt cornered into doing so as they needed to explain why they had either breaks in the medical records or missing records. In relation to accessing both medical and dental care, participants explained that they often needed to discuss with receptionists why they might have either missing or outdated health records. When they were able to access care, a few participants spoke about needing to recap their medical health histories as the clinicians treating them were unable to see information relating to healthcare provided in prison. This created a feeling for the participants that they needed to continuously start from the beginning and lacked continuity in their medical and dental care.

### 3.1 Sharing of power

Power sharing was fundamental to creating a truly co-designed film that accurately represented the lived experiences of the five community returners. Power imbalances between academics and those with a lived experience can lie within hierarchical cultures, further compounded by wider socioeconomic determinants (39). If power imbalances exist, then those with a lived experience may feel unable to express their true views or there is the risk in co-design projects that the views they express are not actioned by the research team. One of the key steps in facilitating the sharing of power was setting clear expectations and ground rules early in the participatory activities, giving responsibility to those taking part in the workshops to form the storyline and content for the film. Whilst a member of the research team facilitated and took accountability for this project, power was shared throughout the co-design project as the film storyline, characters and script were all designed by the community returners who selected which themes they wanted to present in the film. The responsibility and decision as to which themes were outlined in the film was given to the community returners. The themes they chose to portray are shown in Table 1. This expectation was clearly outlined from the start of the co-design process, highlighting that this film was a platform for them to express their experiences and which issues they felt others should be aware of in relation to the oral health of community returners. This power sharing was facilitated by regular communication with participants at all stages of the study, acknowledging their views and contributions, summarising their outputs from each of the sessions, and asking them to make the final revisions of the script. Their names or pseudonyms were included in the credits of the film (after their authorisation) and audience feedback from the open exhibitions of the film were also shared with participants (37).

### 3.2 Including all perspectives and skills

The co-design process required the inclusion of experiences, skills, and beliefs of all those taking part in the project. This was particularly important in this project as we had a range of participants who were experts of their own lived experiences. It was vital that the project was able to portray these varied experiences. For example, participants had different criminal justice histories in relation to the length of sentence they served, the type of prisons they had been in and how long it had been since they had been released. We welcomed this diversity amongst the participants and the trusting relationships formed between the group provided an open and safe space for all individuals to feel comfortable to participate.

After the first workshop it became apparent that the group had two quite different experiences in relation to their oral health journeys through the criminal justice system. Some members of the group had shared that they had poor oral health when they entered the justice system and that oral health had not been *a priori*ty for them. This poor oral health had been further exacerbated by limited access to dental care in prison. On the other hand, for some members of the group had been motivated to look after their oral health prior to being convicted but this motivation waived once they entered prison and lost autonomy over their dietary choices, routines and had restricted access to oral hygiene products. Equally, individuals had varying experiences of accessing care on release, whilst all participants had struggled to access care, one individual was further in their journey and had gone through oral rehabilitation which positively impacted their wider wellbeing. To share all these perspectives, it was decided to have two community returner characters in the film to allow all participants to actively contribute to their storylines and scripts.

To enhance engagement and improve accessibility to participating, several steps were taken to make the workshops a safe and inclusive space. Firstly, during the expectation setting and ground rules section in the first workshop we reinforced how although individuals will have a range of experiences and views, all are equally valid and respected. Secondly, prior to each workshop a document was sent to all individuals via email so they knew what would be discussed and had the opportunity to consider their contributions in advance. Thirdly, in the second workshop the group was split into two groups with each group working on one character that best related to their own lived experience. By individualising the task to the participants and reducing the number of competing options this increased the contributions made by each individual to the final film.

### 3.3 Respecting and valuing different types of knowledge

The ethos of this project from the initiation was that our role as academics was to provide a platform and space to express the views of community returners. As researchers, we had little prior knowledge in this area due to a scarceness of published literature relating to the oral health experiences of community returners. Starting from this perspective allowed the community returner group to lead the creative process through their experiential knowledge. Due to the nature of the project there needed to be an emphasis of all parties being of equal importance to produce the final film alongside true collaborative working. Each party was able to provide an area of expertise that was of equal importance to producing the final film. The research team were required to logistically coordinate the project, workshops and receive funding to support the film. The community returners were essential in providing the content and direction for the film. The community film organisation was involved throughout the process, offering guidance as to what makes an engaging film, conducting the filming itself and editing the film to translate the vision of the community returners into the final output. The actors with lived experience were crucial to conveying the script to an audience and ensuring that the stories depicted were realistic. At multiple stages throughout the project there was a feedback loop to the research team who coordinated the feedback and responded accordingly to ensure that valued opinions were put into practice. An example of this is that the community returners had felt that it would be important for the audience to get a sense of what it feels like to be alone in a cell in prison and how certain cues in the environment can cause a community returner to think back to their time inside. The original cut of the film did not include any sound effects so the views of the community returners were relayed to the film team so that they could add accurate sound effects prior to and during a flashback scene. This proposition was also discussed with the actors on set who offered their own suggestions as to which sound effects could be used to emulate the prison environment.

### 3.4 Reciprocity

The journey that led to the creation of the final film was equally as important as the quality of the final film produced in this project. Reciprocity is the concept that those involved with the project gain something for participating and feel both needed and valued (39). All of those involved with the project should benefit and be recognised for the work that they contribute. This notion was at the heart of the project and considered from the grant writing stage through to the screening of the final film. Reciprocity took many forms in this project. The community returners themselves benefitted by being financially compensated for their time in the form of a voucher. They developed their own connections with other community returners on the project who shared similar lived experiences and were at different stages of their journeys. Additionally, they developed skills around script writing and storytelling. The reception to the film and feedback was relayed to the community returners who participated and has increased their confidence to continue to be involved with similar projects in the future. The contributions of the community returners were acknowledged, and participants were offered the option of being named or using an alias in the credits for the film. They were also invited to the film screening and offered the opportunity to invite friends and family to share their work. The community returners discussed how they valued the opportunity to be involved with the project and were able to contribute their experiences to raise awareness of the oral health challenges they face. Additionally, they felt a sense of achievement at being able to see their contributions depicted in the final film (37).

For the community film organisation this film was their first experience of creating a film that was not a documentary. This film script provided them with a collaborative space to explore a new medium of film and showcase these skills for future projects. This was also their first experience of being part of an oral health project and gave them an appreciation as to the role oral health plays in an individual's wider wellbeing.

The research team were able to benefit from the study in several ways. The completion of the project provides evidence that recruitment of engaged community returners is possible, and they share the opinion that improving oral health inequalities in this population is important. The themes outlined in the film provide avenues for future research endeavours and possible areas to target interventions to improve the oral health of community returners. The use of co-design methodology has developed skills for the research team in power sharing, advocating for others, facilitation and how the arts can be used to convey complex concepts.

### 3.5 Building and maintaining relationships

Underpinning the previous values lies the importance of strong relationships between all parties involved in a co-design project. Successful co-design projects are built on the foundation of compassionate and trusting relationships. Without this key element engagement can be lost, intermittent or participants can feel unable to express their true lived experiences. These relationships precede and transcend the project itself. For example, the recruitment of community returners relied heavily on prior connections that had been made with third sector organisations who support those impacted by the criminal justice system. Building these connections and clearly outlining the expectations of the project, the potential impact of the work and the mutual benefit to community returners meant that they felt confident enough to promote the opportunity to those they support. A facilitator in this was also that when the research team communicated the motivation for conducting this project, third sector groups were able to relate to the need to conduct this work having supported individuals who had spoken about the impact poor oral health has had on their lives. The opportunity being advertised through credible third sector groups was integral to recruiting engaged community returners. It has been cited that community returners have a lack of trust for those in positions of perceived authority (10), therefore, having the opportunity circulated through trusted organisations set the foundations for the project.

Building on this, valuing those involved with the project ensured trusting relationships were sustained. Steps that helped to achieve this were ensuring that expectations were met, and communication was consistent throughout. These relationships have been supported through ensuring that all the individuals involved have been credited for the knowledge they have contributed to the work. From a research team perspective, to develop meaningful relationships with those involved in the project it was important to acknowledge some of the potential barriers including preconceptions, unconscious bias, and power dynamics. Having an awareness and appreciation of this allowed for proactive approaches to overcome these barriers to facilitate honest and open conversations. During the workshops, the research team stepped outside of their day-to-day role and acted simply as facilitators and active listeners for the discussion, allowing the community returners to speak about their experiences. The relationships were further strengthened by delivering on promised outputs, such as emailing community returners following the workshops to thank them for their contributions, distributing anonymised synopses of the workshop discussions, providing the participant vouchers and sharing the final film with them.

## 4 Discussion

Participatory research such as the co-design method used in this film has several benefits including increasing the impact of research and improving the relevance of outcomes produced to service users (40). The process of co-design research also provides a platform for shared learning alongside system partners whilst having a positive emotional impact on those who take part, such as an increase in self-confidence and feelings of pride and accomplishment (41). The preliminary stages of research should focus on determining the research priorities through engagement with target populations to reduce research waste (42). This co-design participatory action research approach to engaging community returners was both successful and impactful. The community returners felt empowered to share their lived experience and ingrain these into the stories of two fictional characters. Furthermore, when they were shown the final film the community returners felt that it encompassed their lived experiences and portrayed their stories accurately.

The themes that they felt were most important to convey in the film were self-care oral health behaviours, access to dental care in prison, a fear of stigmatisation when accessing care on release and a lack of continuity in relation to their health records. Using this co-design approach revealed some themes that have not previously been cited in the literature or considered as research avenues by the academic team conducting the project. An example of this is how when the community returners discussed their experiences of accessing healthcare on release, they spoke about feeling cornered into disclosing their criminal justice history as receptionists often struggled to find their healthcare records. They elaborated on this barrier to care and spoke about how experiencing delays in accessing their health records had impacted their continuity of care and delayed treatment (43).

The community returners were able to reflect upon how their oral health experiences varied throughout their life course. The film presents experiences relating to oral health self-care behaviours prior to incarceration, during prison and then on release. The script touches upon how the prison environment influences oral health both directly through challenging access to dental care but also indirectly due to a lack of autonomy over selecting their diet, accessing oral hygiene aids, and self-managing dental pain. Another consideration raised by the community returners is how on entering prison maintaining the motivation to continue self-care behaviours is difficult. The contributing factors for this reduction in motivation included restricted access oral hygiene aids, mundane daily routines and reduced social contact with family and friends.

### 4.1 Limitations

The co-design process recruited and continually engaged five community returners which is a relatively small number considering approximately 48,000 individuals are released from prison each year in England alone (44). The small sample number reduces the generalisability of the final film as it is possible that the experiences portrayed in the film are not reflective of those experienced by other community returners. However, during the two workshops the participants shared similar thoughts, if a community returner did not personally have lived experience of a topic raised, they were able to relate to it through the stories of other individuals they had encountered through their journeys in the criminal justice system. This validation of comments shared and similarity in themes raised suggests a consensus was reached. Furthermore, the scope of this project is not such that we can comment objectively on the impact the film output has had on the oral health of community returners or stakeholder perspectives. Instead, this engagement project acts as a platform to facilitate future research surrounding the oral health of community returners and explore co-design methodologies to improve their engagement with healthcare research.

One of the challenges of conducting co-design is that for true co-design to exist trusting relationships need to be built which takes time, self-awareness, and emotional investment (45). For a co-design film such as this to be successful it must remain true to the stories it is conveying. From a researcher standpoint, this can be challenging, it requires relinquishing control to participants and embracing uncertainty surrounding the project outputs.

### 4.2 Implications for policy, practice and future research

This co-design project adds to a limited body of evidence surrounding the oral health of community returners and their experiences of accessing care. It provides an indication as to how to actively engage those with a lived experience of the criminal justice system in research that can positively impact their oral health. Since the film has been created it has reached a wide audience including those who work in the criminal justice system or support community returners (37). The film was aired at an open screening held in East London that was attended by academics, dental professionals and those who work in the third sector supporting community returners. Through discussion between the research team and attendees at the screening, the feedback shared from viewers of the film highlighted that they had not previously considered the oral health challenges community returners face and found the film powerfully conveyed these, improving their awareness and allowing them to consider what they can change in their roles to improve the oral health of this group. Alongside raising awareness, the film can be used as an educational and training tool, a catalyst for promoting change in current practice and identifying future research priorities. The film was selected to form part of an annual training session hosted by NHS Education for Scotland which was attended by those working in the national oral health improvement programme for people in prison, Mouth Matters (46). The film allowed practitioners to think beyond supporting oral health in the prison environment alone and consider the lasting influence criminal justice contact can have beyond release. An example of this is that attendees who had an oral health promotion role in their local communities but did not have a prison in their local area considered for the first time that they would have community returners residing in their region. This allowed them to consider how they could support the improvement of oral health in those individuals and help them to access dental care.

The contents described in the film as conveyed through the lived experience of community returners indicate that oral health forms a piece of their larger societal reintegration journey. The community returners have demonstrated how oral health is an important issue for them, influencing wider aspects of their wellbeing such as nutrition and self-confidence. It is hoped that the themes selected by the community returners to be showcased in the film can act as the basis for highlighting future research priorities. The themes indicate where there is the need for development of the evidence base to support improvements in practice and understanding (43). The methodology showcased in this project may also be transferrable to better understanding the oral health experiences of other populations that face exclusion but remain underrepresented in the literature.

## 5 Conclusion

In conclusion, we suggest that co-designing catalyst films is a useful methodology to share the lived experiences of community returners. The methodology utilised in this project allowed community returners to be included in the co-design of a film and demonstrates that they can offer invaluable contributions not previously considered by the research team. This co-design project empowered community returners to use their voices to provide a platform to shape future research through selecting which themes they felt were most important to their oral health experiences.

## Data Availability

The datasets presented in this study can be found in online repositories. The names of the repository/repositories and accession number(s) can be found below: https://www.youtube.com/watch?v=S_UupJJxbaA.
