# Supplementary figures and images for: Co-designing a film showcasing the dental experiences of community returners (ex-offenders)
Source: Front Oral Health. 2025 Jan 6;5:1391438. doi: 10.3389/froh.2024.1391438 (PMC11743650; doi:10.3389/froh.2024.1391438)

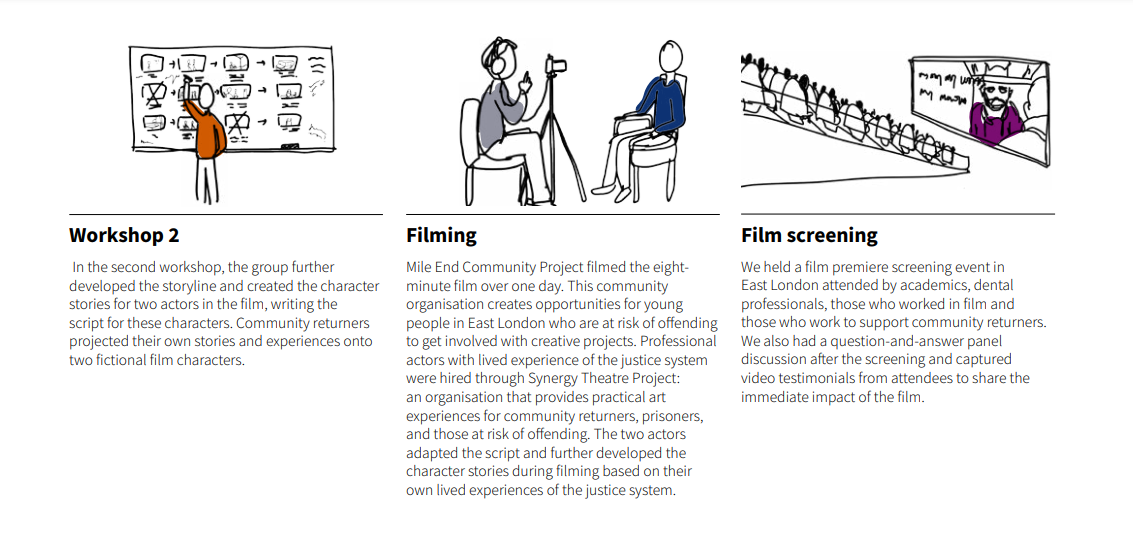

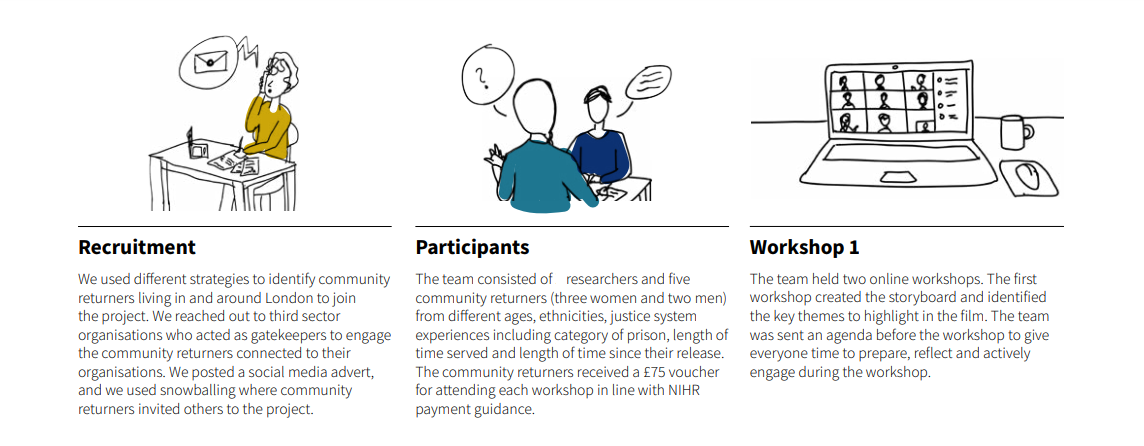

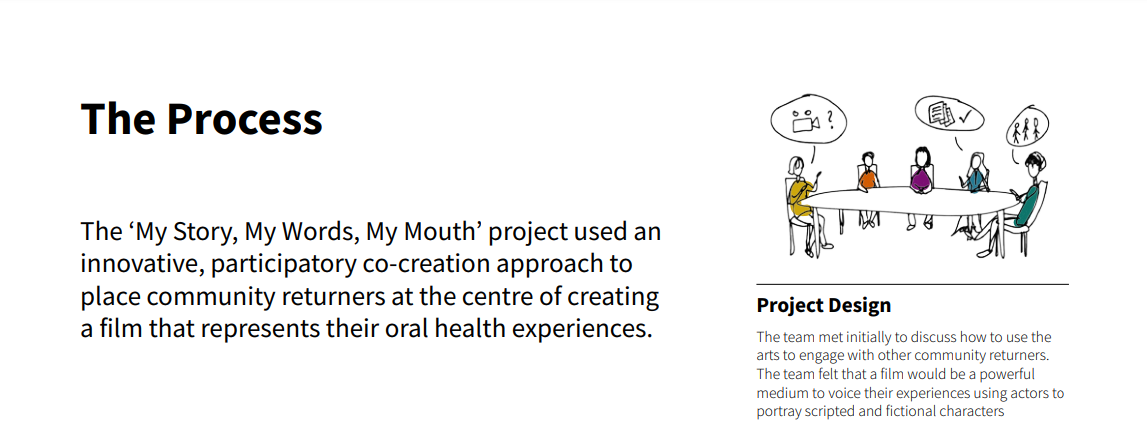

Supplement: Supplementary file 1 [file Table1.docx]
