# Supplementary material for: Co-designing a film showcasing the dental experiences of community returners (ex-offenders)
Source: Front Oral Health. 2025 Jan 6;5:1391438. doi: 10.3389/froh.2024.1391438 (PMC11743650; doi:10.3389/froh.2024.1391438)
Supplement: Supplementary file 2 [file Table2.docx]

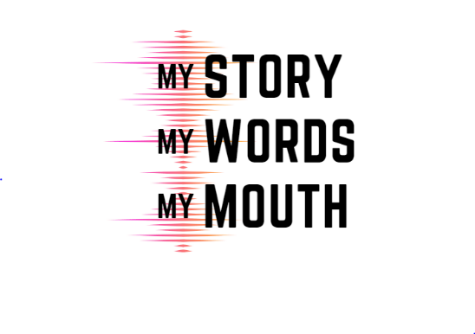


**Workshop One:**

Thank you for signing up to help create a film about your dental experiences. We are looking forward to hearing about your experiences and creating this important film with you.

The first workshop will cover:

- Introductions to the group
- Introduction to the film project with an opportunity to ask questions
- Discussion on the storyline and format for the film
- What words and names to use when speaking about “ex-offenders”
- What to expect in workshop two

To help inspire you I have included a few examples of some films:

1. Mile End Community Films – films made by the organisation that will be making our film: <http://www.mileendcommunityproject.org/films>

Suggested things to think about when watching the films:

- Do you like the use of quotes and words on the screen?
- Do you like the use of animations?
- How many stories shall we tell?
- Shall we use facts on the screen in the film?
- Do you prefer black and white or colour films?
- What makes a film interesting?


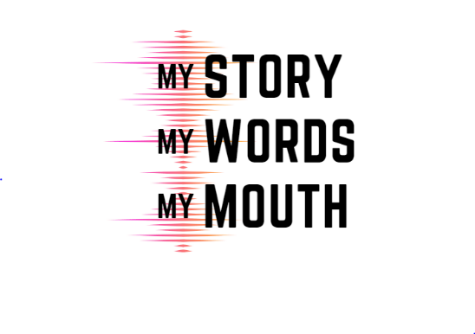


**Workshop Two:**

Thank you for all your great contributions in our first workshop. We are looking forward to working with you again to create the script for this film.

The second workshop will cover:

- A brief recap of what we designed during the first workshop for the film
- Writing the script for the dental waiting room
- Finishing the details of our two characters
- Creating each character’s “flashback” scene and story

As a reminder, we decided at our last workshop that we would have our characters in a dental waiting room. During the waiting room we will hear their thoughts and see their interactions with the dental staff. The waiting room scene will link into flashbacks of their stories and experiences.

To help you I have included the outline of two characters based on our discussions, and we will be splitting into two groups to work on these:

**Male Character** – Very motivated with his health before entering prison. Played squash regularly and looked after himself, sees a dentist every six months for check-ups. Whilst in prison they couldn’t see a dentist due to long waiting lists and the dentist only seeing emergencies. Whilst in prison they weren’t motivated to look after themselves and didn’t have good food options available to choose from. This caused them to have pain from their teeth.

On release they had been taken off the list from their old NHS dentist and struggled to find a dentist. They are worried about the dentist knowing they have been in prison.

**Female Character** – Started using drugs to deal with problems in their life. During this time their teeth was not their priority and they were not looking after themselves.

When they were in prison they had lots of teeth extracted as it is easier for them to extract teeth then to treat them. The appearance of their teeth did not bother them as much as everyone looked the same. When they were released and spent time with other people their age, they were very self-conscious of it. They felt embarrassed about having to tell the dentist why they are so young with no teeth. They had to fight to get a dentist appointment and have their teeth treated but once they did this had a very positive impact on their life.
